# An Overview of Tick-Borne Encephalitis Epidemiology in Endemic Regions of Continental Croatia, 2017–2023

**DOI:** 10.3390/microorganisms12020386

**Published:** 2024-02-13

**Authors:** Tatjana Vilibic-Cavlek, Stjepan Krcmar, Maja Bogdanic, Morana Tomljenovic, Ljubo Barbic, Dobrica Roncevic, Dario Sabadi, Marko Vucelja, Marija Santini, Blazenka Hunjak, Vladimir Stevanovic, Marko Boljfetic, Linda Bjedov, Viktor Masovic, Tanja Potocnik-Hunjadi, Danijela Lakoseljac, Mahmoud Al-Mufleh, Vladimir Savic

**Affiliations:** 1 Department of Virology, Croatian Institute of Public Health, 10000 Zagreb, Croatia; maja.bogdanic@hzjz.hr; 2 School of Medicine, University of Zagreb, 10000 Zagreb, Croatia; marijasantini.ms@gmail.com; 3 Department of Biology, Josip Juraj Strossmayer University of Osijek, 31000 Osijek, Croatia; stjepan@biologija.unios.hr; 4 Department of Epidemiology, Teaching Institute of Public Health of the Primorje-Gorski Kotar County, 51000 Rijeka, Croatia; tomljenovicmorana@gmail.com (M.T.); dobrica.roncevic@zzjzpgz.hr (D.R.); danijela.lakoseljac@zzjzpgz.hr (D.L.); 5 Department of Social Medicine and Epidemiology, Faculty of Medicine, University of Rijeka, 51000 Rijeka, Croatia; 6 Department of Microbiology and Infectious Diseases with Clinic, Faculty of Veterinary Medicine, University of Zagreb, 10000 Zagreb, Croatia; ljubo.barbic@vef.hr (L.B.); vladostevanovic@gmail.com (V.S.); viktor.masovic@gmail.com (V.M.); 7 Department of Public Health, Faculty of Health Studies, University of Rijeka, 51000 Rijeka, Croatia; 8 Department of Infectious Diseases, Clinical Hospital Center Osijek, 31000 Osijek, Croatia; dariocroatia@gmail.com; 9 Medical Faculty, University of Osijek, 31000 Osijek, Croatia; 10 Department of Forest Protection and Wildlife Management, Faculty of Forestry and Wood Technology, University of Zagreb, 10000 Zagreb, Croatia; marko.vucelja@sumfak.unizg.hr (M.V.); marko.boljfetic@sumfak.unizg.hr (M.B.); linda.bjedov@sumfak.unizg.hr (L.B.); 11 University Hospital for Infectious Diseases “Dr. Fran Mihaljevic”, 10000 Zagreb, Croatia; 12 Department of Bacteriology, Croatian Institute of Public Health, 10000 Zagreb, Croatia; blazenka.hunjak@hzjz.hr; 13 Department of Microbiology, University of Applied Health Sciences, 10000 Zagreb, Croatia; 14 Department of Infectious Diseases, General Hospital Varazdin, 42000 Varazdin, Croatia; tanja.potocnik.h@gmail.com; 15 Department of Infectious Diseases, County Hospital Cakovec, 40000 Cakovec, Croatia; mahmoud.almufleh@gmail.com; 16 Poultry Center, Croatian Veterinary Institute, 10000 Zagreb, Croatia

**Keywords:** tick-borne encephalitis virus, epidemiology, *Ixodes ricinus*, Croatia

## Abstract

Tick-borne encephalitis (TBE) represents an important public health problem in Europe. We analyzed the epidemiology of TBE based on data from humans, animals, and *Ixodes ricinus* ticks in endemic regions of continental Croatia. In the period from 2017 to 2023, cerebrospinal fluid (CSF) and serum samples of 684 patients with neuroinvasive diseases, 2240 horse serum samples, and 300 sheep serum samples were tested for TBEV. In addition, 8751 *I. ricinus* ticks were collected. CSF samples were tested using RT-PCR. Serological tests (serum, CSF) were performed using commercial ELISA, with confirmation of cross-reactive samples by a virus neutralization test. Eighty-four autochthonous human TBEV cases were confirmed. The majority of patients were in the age group of 40–69 years (58.3%) with a male predominance (70.2%). TBE showed a bimodal seasonality with a large peak in April–August and a small one in October–November. In addition to humans, TBEV IgG antibodies were found in 12.2% of horses and 9.7% of sheep. Seasonal tick abundance corresponds to the reported number of human infections. Continental Croatia is still an active natural focus of TBE. Continuous monitoring of infections in humans, sentinel animals, and ticks is needed for the implementation of preventive measures.

## 1. Introduction

Tick-borne diseases are becoming an increasing public health problem. Tick-borne encephalitis (TBE) is the most widespread tick-borne viral disease in the Euro-Asia region and the second most common tick-transmitted disease in Europe [1,2].

Tick-borne encephalitis virus (*Orthoflavivirus encephalitidis* virus, according to 2022 ICTV Taxonomy Release; TBEV) is an arbovirus that belongs to the family *Flaviviridae*, genus *Orthoflavivirus*, tick-borne encephalitis serocomplex [3]. In addition to the three main subtypes (European, Far Eastern, and Siberian), two other subtypes (Baikalian and Himalayan) have been described recently [4]. Tick *Ixodes ricinus*, mainly distributed in Europe, is the main vector for TBEV [5]. TBEV infections in humans mostly occur after the bite of an infected tick; however, the number of food-borne TBE infections occurring after consumption of unpasteurized goat milk has been increasing in recent years [6]. Meningitis is observed in about 50% of patients with a neuroinvasive form of TBE, encephalitis in 40%, and myelitis in 5–10% [7]. Meningitis and encephalitis caused by the European subtype are usually milder with a mortality rate of less than 2%. The TBEV Far Eastern subtype causes the most severe disease with mortality of up to 20% [4].

TBE is endemic in 27 European countries. The number of TBEV cases has increased over the past 20 years, counting thousands of human cases every year. The disease is irregularly distributed but continuously expanding in Eastern and Central Europe, as is the spread of *I. ricinus* [8]. Recent records of *I. ricinus* at altitudes over 1000 m above sea level in Norway and 1200 m in Central Europe [9] may increase the risk of TBE in areas that were previously thought to be free of the virus. The majority of TBE cases occur between April and November, coinciding with the period when ticks are most active and the risk of exposure for humans from outdoor activities is highest [10]. Data from several European countries suggest that individuals over 50 years of age have the highest incidence of TBE. However, TBE in children may be significantly underdiagnosed due to its milder clinical course [1]. In addition, the incidence is generally higher in men, which may reflect more frequent exposure to tick bites during work or recreational outdoor activities [11].

Clinical TBEV cases in animals are rare but have been reported in horses [12] and dogs [13]. Other animal species such as goats, sheep, and cattle develop TBEV antibodies without showing clinical signs. However, these species are important as a source of infection in alimentary TBEV infections, as infected animals may excrete the virus in milk. Since the prevalence of TBEV in *I. ricinus* ticks is generally low (less than 1%), seroepidemiological surveys in animals are useful to determine the prevalence and assess the human risk of TBE. Horses, goats, and sheep may be particularly suitable as sentinel hosts because they graze on pastures for long periods each year and are therefore likely to come into contact with many TBEV-infected ticks [14].

In Croatia, TBE was first reported in 1953 near Križevci (Stara Ves, northwestern region) [15]. In addition to this Pannonian focus, the continental foci (Bjelovar, Pakrac, Koprivnica, Karlovac, Varaždin) and several smaller Mediterranean foci near the islands of Zadar, Pula, and Brac have been discovered since 1961 [16]. The disease is endemic in northwestern and eastern continental regions between the Sava and Drava rivers. Endemicity is highest in northwestern counties, with average incidence rates ranging from 3.61 to 6.78 per 100,000 inhabitants [17,18]. In recent years, TBEV has occurred in some areas of the Gorski Kotar, the mountainous region of the central continental part of Croatia [6]. Small foci of TBE have previously been found in the Middle and South Croatian littoral [19]; however, there have been no reported clinical cases in recent years.

Since TBE showed an increasing trend in certain endemic European regions, this study aimed to analyze the epidemiology of TBE, based on data from humans, animals, and ticks in endemic regions of continental Croatia.

## 2. Materials and Methods

### 2.1. Human Sampling and Testing

This study included a total of 684 patients with neuroinvasive disease (febrile headache, meningitis, encephalitis, myelitis) from continental Croatian regions who developed symptoms during the arbovirus transmission season (April–November). Patients were recruited at the infectious disease departments in county hospitals. Viral etiology of neuroinvasive disease was suspected based on predominantly mononuclear pleocytosis, elevated protein levels, and normal glucose levels in the cerebrospinal fluid (CSF) samples. None of the participants reported a history of vaccination against flaviviruses (TBEV or yellow fever). Continental regions were selected based on epidemiological data on TBE incidence in previous seasons [20]. In addition to TBEV, West Nile virus (WNV) and Usutu virus (USUV) testing were also included because of their overlapping clinical symptoms, geographic distribution, and potential serological cross-reactivity.

In all patients, serum and CSF were collected in the acute phase of the disease. CSF samples were tested for TBEV, WNV, and USUV RNA. In addition, serum and CSF samples were tested for TBEV, WNV, and USUV IgM and/or IgG antibodies (Table 1).

Serological testing was performed using commercial enzyme-linked immunosorbent assays (ELISA; Euroimmun, Lübeck, Germany). TBEV IgM/IgG-positive samples were further tested for IgG avidity (Euroimmun, Lübeck, Germany) [24]. The TBEV IgG avidity index (AI) was interpreted as follows: <40% low (acute/recent infection); 40–60% borderline; and >60% high (past infection). Samples with cross-reactive flavivirus antibodies were confirmed using a virus neutralization test in cell culture (VNT) [25,26].

TBE was confirmed in all patients according to the European Center for Disease Prevention and Control (ECDC) clinical (symptoms of the central nervous system inflammation) and laboratory (detection of TBEV IgM and IgG in serum and IgM antibodies in the CSF) criteria [27].

### 2.2. Tick Collection and Identification

**Figure 1:**
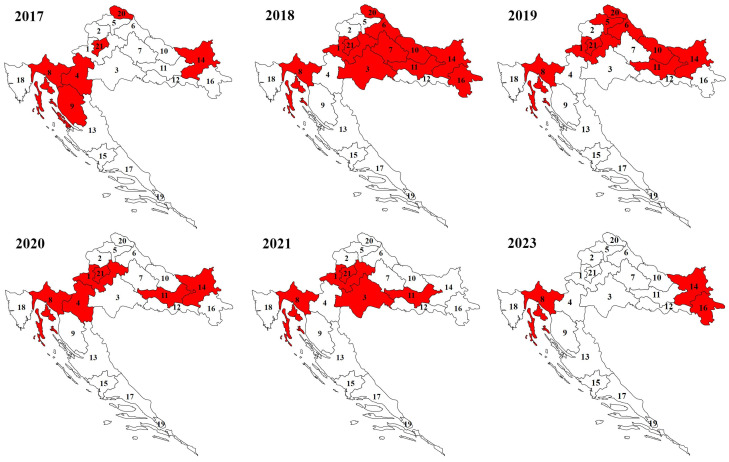
Sampling areas of *Ixodes ricinus* ticks in Continental and Alpine regions of Croatia by year (numbers represent county labels).

In the Continental biogeographic region, ticks were collected in the area of the Medvednica and Papuk mountains, and in the area between the Drava River in the north, the Sava River in the south, and the Danube River in the east. In the Alpine biogeographic region, ticks were mostly collected in the Gorski Kotar area (Figure 1).

Hard ticks were sampled using the dragging–flagging method. The size of the white flannel flag was 1 m^2^. Also, ticks were hand-picked from living domestic and dead wild animals. Continuous samplings of ticks twice a year (spring and autumn) were performed in the area of Zagreb City (21)/Zagreb County (1), Primorje-Gorski Kotar County (8), and Požega-Slavonia County (11) during 2019, 2020, and 2021, and in Karlovac County (4) during 2020 as well as in Osijek-Baranja County (14) and Bjelovar-Bilogora County (7) during 2018. In 2019, ticks were collected from February to August in Osijek-Baranja County (14). During 2023, in Osijek-Baranja County (14) and Primorje-Gorski Kotar County (8), ticks were collected twice per month in the spring and summer months, while in Vukovar-Srijem County (16), ticks were collected only in the spring months. Continuous sampling of ticks was mostly carried out in counties with protected areas such as Nature Parks (Medvednica and Papuk) or Regional Park Mura-Drava and interesting tourist areas of Gorski Kotar, which record a large number of visitors during the spring and autumn months. Furthermore, in all other counties during the study period, ticks were sampled in randomly determined areas at different times throughout the year, mostly during the spring and autumn months. In some counties, ecological studies of tick phenology were not the main goal of this study; therefore, the sampling periods are half-systematic and half-random in these counties. This resulted in differences in the number of tick samplings conducted in the studied areas from year to year. Also, this is partly a consequence of the smaller number of participants in the field samplings of ticks. For this reason, not all counties in the Continental and Alpine regions were covered by tick samplings at the same time. Some ticks were hand-picked from pet animals: dogs *Canis lupus familiaris*, L., 1758, and cats *Felis catus*, L., 1758, and from over-run or hunted wild animals: beech marten *Martes foina* (Erxleben, 1777), hedgehog *Erinaceus roumanicus* Barrett-Hamilton, 1900, Eurasian badger *Meles meles* (L. 1758), the red fox *Vulpes vulpes* (L. 1758), wildcat *Felis silvestris silvestris* (Schreber, 1777), red deer *Cervus elaphus* L. 1758, and wild boar *Sus scrofa* L., 1758. All collected ticks were put into plastic vials and preserved in 96% ethanol.

The identification of ticks was performed using a stereo-microscope (40× magnification) according to available identification keys [28,29].

### 2.3. Animal Sampling and Testing

Animal sampling was conducted among horses (*n* = 2240) and sheep (*n* = 300). Horses were sampled in eight continental counties (four northwestern, three eastern, and one central). Counties were selected based on TBE reports (hot spots) in humans. Sheep were sampled at three locations (Vukovar, Borovo, and Trpinja) in the easternmost Vukovar-Srijem County, selected on the seroprevalence results in horses. Sentinel animals were tested for TBEV IgG antibodies using a commercial ELISA (Immunozym FSME IgG All species, Progen, Heidelberg, Germany). Samples with cross-reactive flavivirus antibodies were confirmed using a VNT, as described above.

### 2.4. Human, Animal, and Tick Sampling Timeline

**Figure 2:**
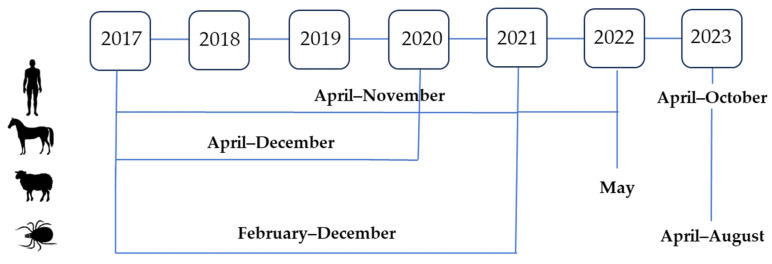
Sampling timeline in humans, animals, and ticks, 2017–2023.

The sampling timeline is presented in Figure 2. Human sampling was conducted from April to November 2017–2022 and from April to October 2023. Horses were sampled from April to December 2017–2020. Sheep were sampled in May 2022. Tick samplings were mostly carried out from the beginning of February to mid-December in the period from 2017 to 2021 and from April to August 2023.

### 2.5. Statistical Analysis

The differences in TBEV seropositivity according to patient demographic characteristics were compared using a Chi-square test. Statistical analysis was performed using the Web Social Science Statistics program (https://www.socscistatistics.com/, accessed on 18 January 2024).

## 3. Results

### 3.1. Geographic and Seasonal Distribution of Tick-Borne Encephalitis in Humans

**Figure 3:**
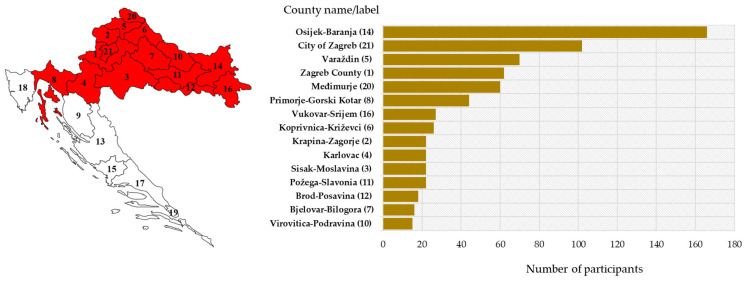
Geographic distribution of the study participants: sampling area (red-shadowed) with county labels (**left**) and number of participants by county (**right**).

The majority of patients were from eastern (Osijek-Baranja County) and northwestern counties (City of Zagreb/Zagreb County; Varaždin County and Međimurje County) (Figure 3).

During the testing period, TBE was confirmed in 87/684 (12.7%) patients who presented with neuroinvasive disease: 84 (12.3%) cases were autochthonous, and 3 cases were imported from Germany, the Czech Republic, and Sweden.

**Figure 4:**
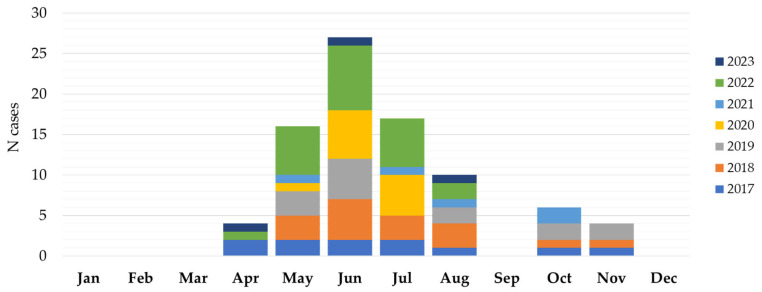
Seasonal distribution of tick-borne encephalitis in Croatia, 2017–2023.

The seasonal distribution of TBEV cases is presented in Figure 4. TBE in Croatia showed a bimodal seasonality with two peaks: a larger one in the spring and summer months (April–August) and a smaller one in autumn (October–November). The largest number of patients was reported from May to July (59; 70.2% cases), with a peak in June (26; 30.9% cases). In June 2019, a small outbreak (six cases) following the consumption of raw goat milk was detected in Gorski Kotar. Clustering was also observed in the same but wider area in June and July 2022 (10 cases).

**Figure 5:**
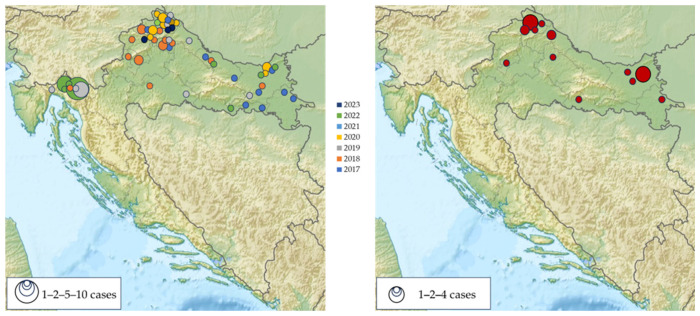
Geographic distribution of acute tick-borne encephalitis cases in Croatia, 2017–2023 (**left**); IgG-seropositive participants (**right**).

The majority of TBE patients (37; 44.0%) were recorded in six north-western counties, followed by two easternmost counties (11; 13.1%). Two clusters were reported in the Gorski Kotar region (2019, 2022). Most TBEV IgG-seropositive participants were detected in the same geographic regions (Figure 5).

**Figure 6:**
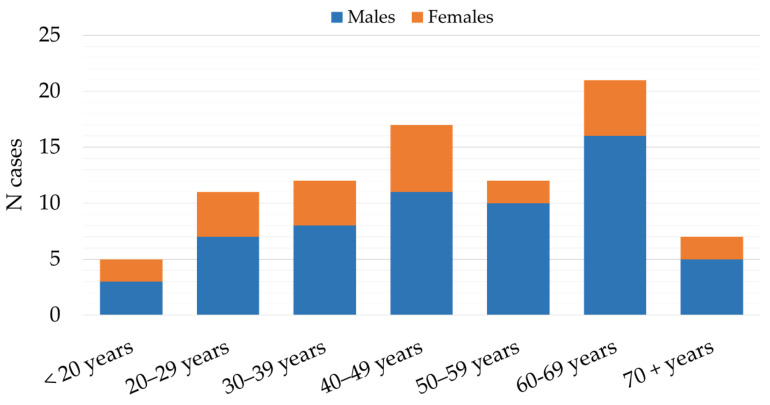
Age and gender distribution of patients with tick-borne encephalitis in Croatia, 2017–2023.

The patients with TBE were mostly males (59; 70.2%), with a male-to-female ratio of 2.4:1. Males predominated in all age groups with ratios ranging from 1.3:1 (<20 years age group) to 5:1 (50–59-year age group). Most infections were detected in patients aged 40–69 years (49; 58.3%). The lowest number of cases were detected in the age groups of 70+ years (7; 8.3%) and < 20 years (5; 5.9%) (Figure 6).

**Figure 7:**
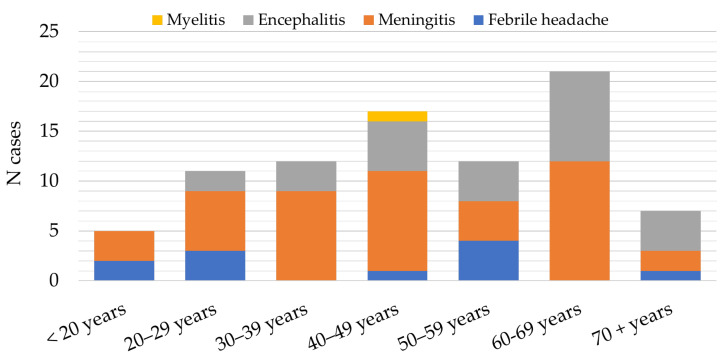
Distribution of patients with tick-borne encephalitis in Croatia according to clinical presentation, 2017–2023.

The main clinical presentations in patients with confirmed TBE were meningitis (46; 54.8%) and encephalitis (26; 30.9%). Eleven patients (13.1%) presented with febrile headache and one (1.2%) with meningoencephalomyelitis. Meningitis was the most common clinical presentation in patients less than 50 years (28; 60.9%), while encephalitis was observed more frequently in the age groups above 60 years (17; 65.4%) (Figure 7).

In addition to acute infections, 20 (2.1%; 95%CI = 1.1–3.4) patients showed previous TBEV exposure (IgG seropositive). Analyzing the TBEV IgG seroprevalence (Table 2), there was no difference in the seropositivity between males and females (2.6% vs. 3.6%). In addition, age-related differences in seropositivity were not significant. In the age group up to 29 years, the IgG seroprevalence was 3.7%. The seropositivity in the age groups of 30–49 years and 50+ years was 3.2% and 2.5%, respectively.

### 3.2. Geographic and Seasonal Distribution of Ixodes ricinus Ticks

**Figure 8:**
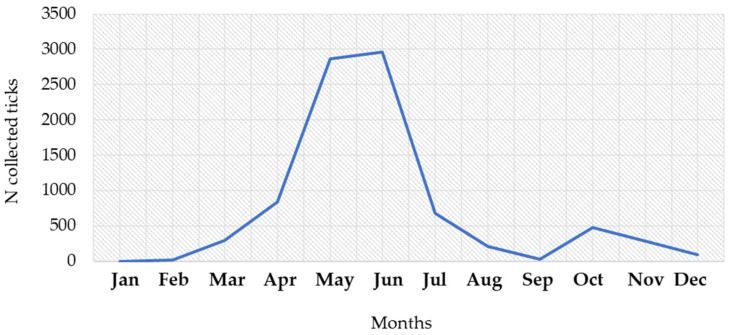
Seasonal distribution of Ixodes ricinus ticks, 2017–2023.

During the study period, a total of 8751 *I. ricinus* ticks were collected. The number of collected ticks by month is presented in Figure 8. The largest number of ticks were found from April to July (7354; 84.0%), with a peak in June (2965; 33.9%) and May (2866; 32.7%). In addition, 754 (8.6%) of the collected ticks were found in October–November (Supplementary Table S1). The seasonal tick abundance corresponds to the number of reported human infections.

### 3.3. Tick-Borne Encephalitis in Animals

**Figure 9:**
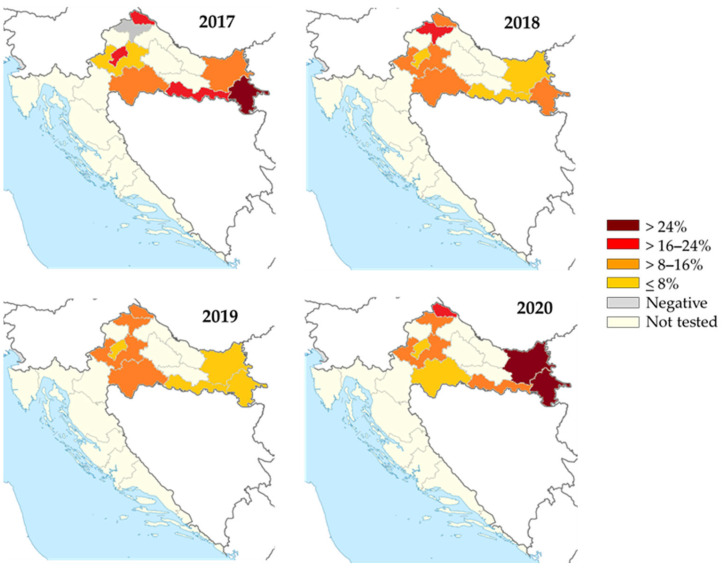
Geographic distribution of tick-borne encephalitis IgG-seropositive horses, 2017–2020. The colors represent seroprevalence rates (%).

TBEV IgG antibodies were detected in 273/2240 (12.2%; 95%CI = 10.9–13.6%) horses. The spatial and temporal seroprevalence rates of TBEV in horses are presented in Figure 9. The overall seroprevalence rates varied significantly between years (*p* < 0.001) as follows: 14.3% (80/560; 95%CI = 11.5–17.5) in 2017, 10.0% (56/560; 95%CI = 7.6–12.8) in 2018, 7.3% (41/560; 95%CI = 5.3–9.8) in 2019, and 17.1% (96/569; 95%CI = 14.1–20.5) in 2020. The seropositivity was highest in the eastmost (140/840; 16.7%, 95%CI = 14.2–19.4%) and northwestern counties (126/1094; 11.5%, 95%CI = 9.7–13.6%). Analyzing the seropositivity by counties, the seroprevalence rates were 0–28.5% in 2017, 4.3–20.5% in 2018, 2.8–14.4% in 2019, and 5.5–35.7% in 2020.

**Figure 10:**
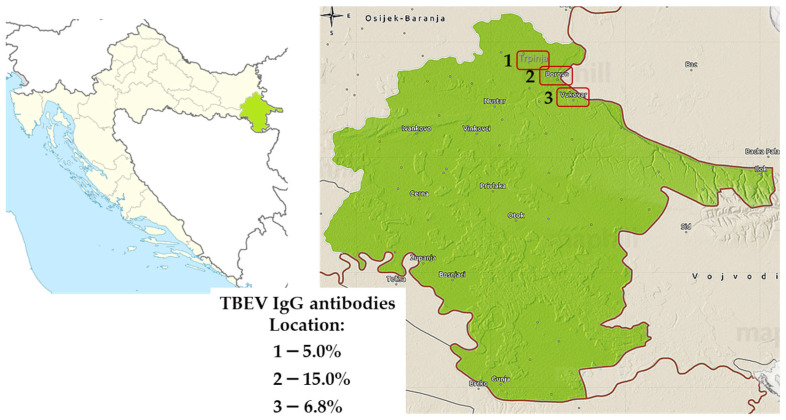
Distribution of tick-borne encephalitis virus IgG-seropositive sheep, 2022. Seroprevalence rates (%) are presented for three locations: Trpinja (1), Borovo (2), and Vukovar (3).

Among the 300 tested sheep in 2022, 29 (9.7%, 95%CI = 6.6–13.6) were seropositive to TBEV. According to the geographic location, seroprevalence rates varied from 5.0% (Trpinja) to 15.0% (Vukovar) (Figure 10).

## 4. Discussion

The incidence of tick-borne diseases has increased dramatically (5–15% annually) over the past two decades in Europe and other parts of the world. In addition, cases were detected in countries where TBE has not yet been reported [30,31]. In a large European study that included the period from 2012 to 2020, 19 countries reported 24,974 TBE cases. Lithuania, Latvia, and Estonia had the highest reporting rates [1]. The continuing increase in the number of TBEV strains detected in *I. ricinus* in Europe and the westward spread of *I. persulcatus* and the more virulent TBEV European and Siberian strains is of great concern [4,5].

In this study, TBE was detected in 12.3% of tested Croatian patients with neuroinvasive disease. Infections were detected in all previously known endemic regions of continental Croatia, while in 2019, a small outbreak of alimentary TBE was recorded in a new micro-focus in the Gorski Kotar region. In addition, a clustering of TBE was observed in the same, but wider area in 2022.

TBE in Croatia showed a bimodal seasonal variation, similar to those reported in other European countries [1]. Cases were detected during the arbovirus transmission season (i.e., April–November) with a larger peak from May to July and a smaller one in October and November. In the rest of Europe, the transmission of TBE was found to be year-round with most cases (98.8%) occurring between April and November. A bimodal distribution of autochthonous cases was observed in all analyzed years except 2012 and 2016, with a first major peak around the first week of July, and a second peak, usually smaller, at the end of September [1]. While in Europe, the most frequently reported month of disease onset was July, in Croatia, it was June, with 30.9% of the recorded cases. However, there were some seasonal differences observed between Northern and Southern Europe. In central and southern countries, the main peak was observed earlier (June–July), compared with northern countries (July–August). In addition, the transmission remained high throughout the summer with a decrease in October in northern countries, while the number of infections decreased significantly in August in central/southern countries [1].

Similar to the results of our study (70.2% male patients, male-to-female ratio of 2.4:1), the data from European countries showed that TBE cases were predominantly male (59.5%, male-to-female ratio of 1.5:1) [1,32]. Recent studies from Scandinavia found that one factor contributing to the increased prevalence in males may be that men and women apply protective measures against tick bites differently. Women are well-informed and more aware of tick-borne diseases and therefore are more likely to use protective measures [33,34,35]. Gender differences in occupations and leisure activities resulting in differential exposure to ticks as well as biological factors may also contribute to this gender difference [1].

Analyzing the distribution of acute TBE cases according to age, more than half of the Croatian patients (58.3%) were in the age group of 40 to 69 years, while the lowest proportion of patients was in the youngest (<20 years) and oldest (>70 years) age groups (5.9% and 8.3%, respectively). Similarly, the mean incidence of TBE was highest in the same age group in a German study (2001–2018) [36]. In addition, data from other European countries showed that incidence is typically low in children and increases with age, with the majority of TBE cases reported in the age group of 45–64 years [32].

In addition to the TBEV subtype, published data suggest a relationship between patient age and TBE severity. Generally, the clinical course of the disease is milder in children than in adults, with meningitis as a predominant form occurring in about 70% of cases [10]. In a study from Lithuania, a highly endemic country for TBE, the highest rate of severe cases (41.2%) was in the 70–79-year age group, with 23.7% of patients presenting meningoencephalomyelitis [37]. In our study, meningitis was the most common clinical manifestation detected in 60.9% of patients younger than 50 years, whereas encephalitis was more commonly observed in the age group of 50 years and older (65.4%). A similar distribution of clinical TBE presentations was recorded in Germany. Patients with meningitis were significantly younger than patients with meningoencephalitis and were less often treated in intensive care units [38]. Older people are more susceptible to symptomatic TBE infections and severe illness due to a diminished immune response [31]. Moreover, antibody responses to TBEV vaccination decline with age, so vaccination failure is more common in older individuals [39].

In addition to acute cases, 2.1% of patients included in our study showed evidence of previous TBE exposure (IgG-seropositive). In contrast to male predominance in acute TBE cases, the IgG seropositivity did not differ between males (2.6%) and females (3.6%). Regarding age, no significant differences in seropositivity were observed between age groups (2.5–3.7%).

Most domestic animals are considered to be useful sentinels for TBE risk in humans [40]. Although there are only a few reports of clinical TBE infections in horses, seroprevalence studies suggest that infections in horses are common in TBE-endemic areas [41,42]. Infected animals with no apparent disease play a role in the maintenance and spread of TBEV either as the tick harbor or diseased dead-end hosts [43]. Some seroprevalence studies in horses conducted since 2000 showed TBE seropositivity of up to 26.1% in Austria, 0.8–23.4% in Germany [43,44,45], 3.45% in Slovakia [46], and 3.1% in Spain [47]. A very high seroprevalence rate of 37.5% and 3.9% TBEV viremic horses were detected in Lithuania [48]. Among domestic animals, sheep are very susceptible to TBEV infection; for example, in Poland TBEV was recorded in 22.2% of sheep’s milk [43].

Seroprevalence studies of TBEV in horses in Croatia are scarce. In 2016, randomly selected horse serum samples from six Croatian counties (five continental and one at the Croatian littoral) were tested for TBEV IgG antibodies. The highest seropositivity was detected in northwestern regions, ranging from 4.5% to 23.6% [49]. In the present study, the highest seroprevalence in horses was observed in the eastern (16.7%) and northwestern regions (11.5%). Significant differences in the TBEV seroprevalence in horses may be related to different risks in a particular season, but also the result of different methods used to keep animals in the same area (horses on pasture or horses in stables), which needs to be further investigated. In addition, 9.7% of TBEV-seropositive sheep were recorded in the easternmost Vukovar-Srijem County. The detection of seropositive animals confirmed their role as sentinels in TBEV monitoring. Using sentinels, the natural foci of TBEV can be identified and more in-depth analysis can be initiated, including tick studies [50].

*Ixodes ricinus* is the main vector of TBE in most of Europe. Some studies showed that in different parts of Europe, *I. ricinus* density exceeded 100 per 100 m^2^ [51]. However, in contrast to other tick-borne pathogens, the prevalence of TBEV in the *I. ricinus* population is generally very low (0.1–0.5%) [4], although, in some endemic regions, the prevalence of TBEV in ticks reaches 20–40% [52]. *Ixodes ricinus* is a very adaptable species and can exhibit different seasonal activity even in adjacent parts of its geographical range [53]. The seasonal dynamic of *I. ricinus* in central Europe is bimodal, with an early to midsummer (May–July) peak and an autumn peak in September [54]. In our study, the autumn peak was noticed in October, while the first peak was recorded in June. Recently, a similar early summer peak of activity for *I. ricinus* was recorded in Romania [55].

This study has some limitations that need to be addressed. The selected counties for human and animal sampling were not the same each year. This study covered counties where TBEV was known to be endemic, and the selection of regions was based on epidemiological data from previous transmission seasons and included only counties with high TBE prevalence. These limitations may, at least partly, influence the differences in the TBE prevalence and should be taken into account when interpreting the results.

## 5. Conclusions

In Croatia, the natural foci of TBEV are still active in the northwestern and northeastern regions. Neuroinvasive human TBE infections were continuously recorded during the study period (2017–2023). Two new natural micro-foci have emerged in the central mountainous region south of the Sava River since 2019. The majority of TBE infections were detected in the ages of 40 to 69 years (58.3%), with male predominance (70.2%). In addition to human cases, the high prevalence of TBEV IgG antibodies in horses and sheep indicates that continental Croatia is still an active natural focus of TBE. Further continuous studies in patients with neuroinvasive disease, asymptomatic individuals, and sentinel animals as well as tick distribution and TBEV detection in ticks are needed to monitor the dynamics of TBE and define risk areas to protect human health.

## Figures and Tables

**Table 1:** Tests used for the detection of neuroinvasive flaviviruses.

Virus	ELISA/VNT	RT-PCR Protocols	
TBEV	ELISA IgM: Ratio < 0.8 negative; 8.8–1.1 borderline; >1.1 positive ELISA IgG: RU/mL < 16 negative; 16–22 borderline; >22 positive VNT Titer ≥ 10 positive	FP: GGG CGG TTC TTG TTC TCC RP: ACA CAT CAC CTC CTT GTC AGA CT Probe: FAM-TGA GCC ACC ATC ACC CAG ACA CA-TAMRA	Schweiger and Cassinotti [21]
WNV		FP: AAG TTG AGT AGA CGG TGC TG RP: AGA CGG TTC TGA GGG CTT AC Probe: FAM-CAA CCC CAG GAG GAC TGG-TAMRA	Tang et al. [22]
USUV		FP: CAA AGC TGG ACA GAC ATC CCT-TAC RP: CGT AGA TGT TTT CAG CCC ACGT Probe: FAM-AAG ACA TAT GGT GTG GAA GCC TGA TAG GCA-TAMRA	Nikolay et al. [23]

RU = relative units; VNT = virus neutralization test; TBEV = tick-borne encephalitis virus; WNV = West Nile virus; USUV = Usutu virus.

**Table 2:** Tick-borne encephalitis virus IgG seroprevalence according to demographic characteristics.

Characteristic	N (%) Tested	TBEV IgG Prevalence		
		N (%) Positive	95%CI	*p*
Gender				
Male	491 (71.8)	13 (2.6)	1.4–4.5	0.493
Female	193 (28.2)	7 (3.6)	1.5–7.3	
Age group				
≤29 years	162 (23.7)	6 (3.7)	1.4–7.8	
30–49 years	157 (22.9)	5 (3.2)	1.0–7.3	0.622
50+ years	365 (53.4)	9 (2.5)	1.1–4.6	

TBEV = tick-borne encephalitis virus; CI = confidence interval.

## Data Availability

The data are contained within this article and Supplementary Materials.

## Supplementary Materials

The following supporting information can be downloaded at: https://www.mdpi.com/article/10.3390/microorganisms12020386/s1, Table S1: Seasonal distribution of *Ixodes ricinus* ticks.
