# Supplementary material for: An Overview of Tick-Borne Encephalitis Epidemiology in Endemic Regions of Continental Croatia, 2017–2023
Source: Microorganisms. 2024 Feb 13;12(2):386. doi: 10.3390/microorganisms12020386 (PMC10891638; doi:10.3390/microorganisms12020386)
Supplement: Supplementary file 1 [file microorganisms-12-00386-s001.zip › microorganisms-2753505-supplementary.pdf]

Table S1. Seasonal distribution of *Ixodes ricinus* ticks

**County (County number)**	**Months**										
	**II**	**III**	**IV**	**V**	**VI**	**VII**	**VIII**	**IX**	**X**	**XI**	**XII**
Zagreb County (20)			109	164	43				10		
Sisak-Moslavina (3)				7				2	11	10	13
Karlovac (4)		17	16	13	6	38	22	15	17		
Varaždin (5)					118						
Koprivnica-Križevci (6)			56		52	13			8		
Bjelovar-Bilogora(7)		6	5	57		48	2	9		7	
Primorje-Gorski Kotar (8)		12	20	1199	2095	470	132		167	3	
Lika-Senj (9)											2
Virovitica-Podravina (10)				13	20				5		
Požega-Slavonia (11)					320	19			42	22	
Osijek-Baranja (14)	18	177	368	693	92	86	55		61	242	76
Vukovar-Srijem (16)		85	45	21		5					
Međimurje (20)					193				3		
Zagreb City (21)			223	699	26	2			146		
Total N	18	297	842	2866	2965	681	211	26	470	284	91
